# The activity of a yeast Family 16 methyltransferase, Efm2, is affected by a conserved tryptophan and its N‐terminal region

**DOI:** 10.1002/2211-5463.12153

**Published:** 2016-11-16

**Authors:** Joshua J. Hamey, Gene Hart‐Smith, Melissa A. Erce, Marc R. Wilkins

**Affiliations:** ^1^ Systems Biology InitiativeSchool of Biotechnology and Biomolecular SciencesUniversity of New South WalesSydneyAustralia

**Keywords:** lysine methylation, methyltransferase, *Saccharomyces cerevisiae*

## Abstract

The Family 16 methyltransferases are a group of eukaryotic nonhistone protein methyltransferases. Sixteen of these have recently been described in yeast and human, but little is known about their sequence and structural features. Here we investigate one of these methyltransferases, *Saccharomyces cerevisiae* elongation factor methyltransferase 2 (Efm2), by site‐directed mutagenesis and truncation. We show that an active site‐associated tryptophan, invariant in Family 16 methyltransferases and at position 222 in Efm2, is important for methyltransferase activity. A second highly conserved tryptophan, at position 318 in Efm2, is likely involved in S‐adenosyl methionine binding but is of lesser consequence for catalysis. By truncation analysis, we show that the N‐terminal 50–200 amino acids of Efm2 are critical for its methyltransferase activity. As N‐terminal regions are variable among Family 16 methyltransferases, this suggests a possible role in determining substrate specificity. This is consistent with recently solved structures that show the core of Family 16 methyltransferases to be near‐identical but the N termini to be structurally quite different. Finally, we show that Efm2 can exist as an oligomer but that its N terminus is not necessary for oligomerisation to occur.

**Abbreviations**
AdoMet: S‐adenosyl methionine
eEF2: eukaryotic elongation factor 2
Efm2: elongation factor methyltransferase 2
MTF16: methyltransferase Family 16

Protein methylation is a widespread post‐translational modification in the eukaryotic cell. While the methylation of histones has been widely characterised, methylation of nonhistone proteins has gained increased attention due to its roles in cellular signalling and disease 1, 2, 3, 4, 5, 6, 7. In order to expand the knowledge of nonhistone methylation, there have been many recent proteome‐scale studies to discover protein methylation sites 8, 9, 10, 11, 12, 13. However, the function of the vast majority of these sites remains elusive. One critical step in understanding the function of protein methylation is to identify and characterise the methyltransferases that catalyse it.

Protein methyltransferases can be separated into two main groups based on their methyltransferase domain: SET domain methyltransferases and seven‐beta‐strand methyltransferases, also called Class I methyltransferases 6. Additionally, there is one known protein methyltransferase with the SPOUT fold 14. All SET domain methyltransferases are protein methyltransferases that are specific to lysine 15, while seven‐beta‐strand methyltransferases are more diverse in their substrates. Seven‐beta‐strand methyltransferases, which make up the majority of all methyltransferases, are known to methylate DNA, RNA, metabolites and other small molecules, as well as proteins 16. It is therefore difficult to predict the substrate specificity of seven‐beta‐strand methyltransferases. Nonetheless, there have been many attempts to predict the substrate specificity of yeast methyltransferases based on features beyond their core fold 17, 18.

Recently, a subclass of seven‐beta‐strand methyltransferases has been discovered which has, so far, proven to be protein‐specific: the Family 16 group of methyltransferases. There are 16 protein methyltransferases in *Saccharomyces cerevisiae* and human which belong to this family, 12 of which have described substrates (Table 1). All members appear to exclusively methylate nonhistone proteins. The *S. cerevisiae* members target translation‐associated proteins. Efm2 and Efm3 methylate eukaryotic translation elongation factor 2 (eEF2), Efm6 and Efm7 methylate eukaryotic translation elongation factor 1A (eEF1A), while Rkm5 and Hpm1 methylate ribosomal proteins RPL1A/B and RPL3 19, 20, 21, 22, 23, 24, 25, 26. The human members target more diverse substrates: CaM‐KMT methylates calmodulin, VCP‐KMT methylates the valosin‐containing protein (VCP), HSPA‐KMT methylates a number of 70 kDa heat shock proteins, METTL22 methylates KIN17, ETFB‐KMT methylates electron transfer flavoprotein subunit beta and eEF2‐KMT, the orthologue of Efm3, methylates eEF2 21, 27, 28, 29, 30, 31, 32, 33. Interestingly, no other substrates have been described for each of these methyltransferases, despite attempts to find more 21, 28, 30, 34. The Family 16 methyltransferases therefore have very restricted substrate specificity. This is probably due to the fact that they recognise three‐dimensional aspects of their substrates and not just sequence motifs 24, 28, 35. It is not yet known, however, what aspect of the Family 16 methyltransferases determines their specificity.

**Table 1:** Family 16 methyltransferases in yeast and human

Name	Organism	Substrate and site	Methylation degree	UniProt accession	Ref(s)
Efm2	Yeast	eEF2‐K613	Di	P38347	19, 20
Efm3	Yeast	eEF2‐K509	Tri	P47163	20, 21, 22
Efm6	Yeast	eEF1A‐K390	Mono	P53970	23
Efm7	Yeast	eEF1A‐G2/K3	Tri/Di	Q05874	24
Rkm5	Yeast	RPL1A/B‐K47	Mono	Q12367	25
Hpm1	Yeast	RPL3‐H243	Mono	P40481	26
VCP‐KMT	Human	VCP‐K315	Tri	Q9H867	28, 29
HSPA‐KMT	Human	Hsp70s: HSPA1‐K561, HSPA5‐K585, HSPA8‐K561	Tri	Q8WXB1	29, 30
METTL22	Human	KIN17‐K135	Tri	Q9BUU2	29, 31
eEF2‐KMT	Human	eEF2‐K525	Tri	Q96G04	21
CaM‐KMT	Human	Calmodulin‐K116	Tri	Q7Z624	27
ETFB‐KMT	Human	ETFB‐K200/K203	Tri/Tri	Q8IXQ9	32, 33
METTL18	Human	–	–	O95568	–
METTL21B	Human	–	–	Q96AZ1	–
METTL21C	Human	–	–	Q5VZV1	–
METTL23	Human	–	–	Q86XA0	–

All Family 16 methyltransferases contain a [D/E]XX[Y/F] motif. This is important for methyltransferase activity, as evidenced, for example, by loss of activity of VCP‐KMT when the aspartate is mutated 28. Besides this, however, there have been no functional studies into the sequence features of Family 16 methyltransferases. We previously noted the presence of two highly conserved tryptophans in Efm2 19. Here, we use mutagenesis and structural models of Efm2 to show that one of these residues is important for methyltransferase activity, while the other is of lesser consequence. We also show that an extended N‐terminal region of Efm2, of about 200 residues, is also critical for its methyltransferase activity. We suggest that it may be involved in binding its substrate eEF2, and that this highly variable region among Family 16 methyltransferases may be responsible for their substrate specificity.

## Materials and methods

### Bioinformatic analysis

All yeast and human Family 16 methyltransferases were aligned using Clustal Omega 36. This alignment was then used to generate a sequence logo using Web logo 37. The domain structures of Family 16 proteins were visualised using CDvist 38. Efm2 was modelled with Swiss‐Model 39 based on the structure of METTL21D (PDB ID: 4LG1) and disorder predicted by pondr‐fit 40. Structures of METTL21A‐D were acquired from the RCSB Protein Data Bank (www.rcsb.org) 41 with IDs of PDB: 4LEC, 4QPN, 4MTL and 4LG1.

### Expression and purification of eEF2, Efm2 and mutant Efm2

N‐terminal truncation mutants of Efm2 were generated by site‐directed ligase‐independent mutagenesis (SLIM) 42. Tryptophan‐to‐phenylalanine mutations in Efm2 were generated by site‐directed mutagenesis 43. Efm2 and mutated Efm2 were overexpressed and purified from *Escherichia coli* (Rosetta DE3), while eEF2 was overexpressed and purified from a ΔEFM2 yeast strain, according to previous methods 20.

### *In vitro* methylation

*In vitro* methylation reactions were performed and analysed by SDS/PAGE and immunoblotting according to previous methods 20. Briefly, eEF2 was incubated with Efm2 (wild‐type or mutant) in the presence of 50 μm S‐adenosyl methionine (AdoMet) in 1× *in vitro* methylation buffer (50 mm HEPES‐KOH, 20 mm NaCl, 1 mm EDTA, pH 7.4) at 30 °C for 1 h, unless otherwise indicated. The antibodies used for immunoblotting were the methylated lysine antibody ab7315 (1 : 1000 dilution; Abcam, Cambridge, UK) and anti‐PentaHis HRP‐conjugated antibody (1 : 5000 dilution; Qiagen, Hilden, Germany, 34460). Ab7315 does not recognise the K509 trimethylation site on eEF2 (see negative controls in Figs 3 and 4), which is catalysed by Efm3 20, 21, 22.

### Mass spectrometry

Samples were analysed on an Orbitrap Velos Pro (Thermo Fisher Scientific, Waltham, MA, USA) according to previous methods 44. Extracted ion chromatograms (XICs) for peptides were obtained using thermo xcalibur qual browser 2.2 SP1.48 by setting mass windows of ±10 ppm of the relevant *m*/*z* value, and applying a scan filter to only analyse MS1 scans. Methyl‐peptide identities were confirmed by comparison with a synthetic equivalent (see Fig. S1 for representative spectra), as done previously 45.

### *In vitro* crosslinking

Efm2 and N‐terminal truncation mutants, in 25 mm sodium phosphate buffer, 100 mm NaCl, 20% (v/v) glycerol, 5 mm β‐mercaptoethanol, 0.2 m triethanolamine, were crosslinked with 330 μm dimethyl pimelimidate (Thermo Fisher Scientific) for 2 h at room temperature. For the 60‐min time course assay of Efm2 crosslinking, 410 μm dimethyl pimelimidate was added instead. SDS was added to a final concentration of 1% prior to addition of the crosslinker for a negative control. SDS sample buffer was added to quench the crosslinking reaction and samples were analysed by SDS/PAGE and immunoblotting as described above.

## Results

### Family 16 methyltransferases show conservation of two key tryptophans and variable N‐terminal regions

**Figure 1:**
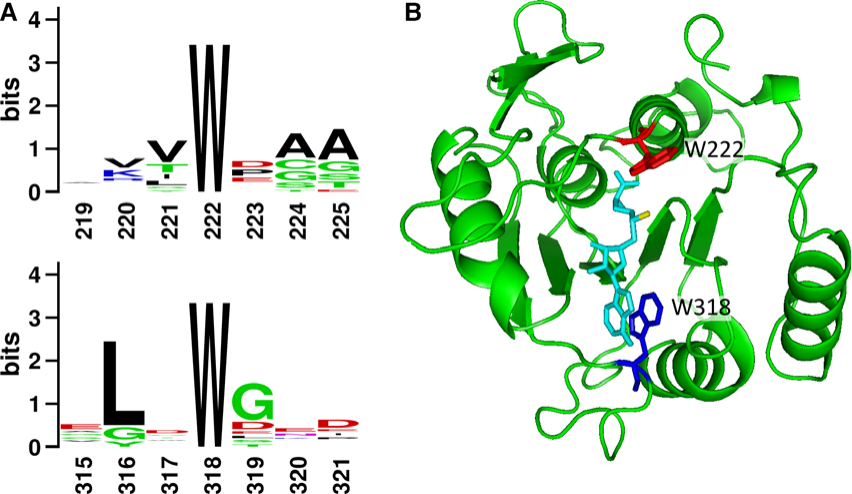
Two highly conserved tryptophans are situated near the location of AdoMet binding in Family 16 methyltransferases. (A) Sequence logo for all Family 16 methyltransferases from yeast and human, showing the relative conservation of W222 (top) and W318 (bottom) numbered relative to Efm2. The *y*‐axis shows the total conservation of each position (total height of each stack) and the relative conservation of each amino acid (relative heights of each letter). Maximum conservation is the maximum entropy for amino acid sequences (log_2_ of 20 amino acids = 4.3). W222 showed 100% conservation, while W318 was only absent in ETFB‐KMT. The full sequence logo is shown in Fig. S2. (B) The predicted structure of residues 192–406 Efm2 by homology modelling based upon the structure of VCP‐KMT (PDB ID: 4LG1). Efm2 is shown as a ribbon structure in green, with tryptophans 222 and 318 shown as stick structures in red and blue respectively; AdoMet is shown as a stick structure in cyan, with the donated methyl group shown in yellow. Visualised in pymol (The PyMOL Molecular Graphics System, Version 1.3 Schrödinger, LLC, New York, NY, USA).

In order to better understand the potential significance of tryptophans W222 and W318 in Efm2, their conservation was investigated by aligning all yeast and human Family 16 methyltransferases and generating a sequence logo from this alignment (Fig. 1A, see Fig. S2 for entire sequence logo). W222 showed 100% conservation, while W318 was absent only in ETFB‐KMT (Fig. 1A). We then investigated the structural contexts of both these tryptophans in a homology‐predicted model of Efm2. Strikingly, both tryptophans are positioned at the location of methyl‐donor AdoMet binding (Fig. 1B). W222 is positioned near the active site, as has been noted previously 22, while W318 is positioned adjacent to the imidazole ring of AdoMet, and may therefore stabilise the binding of AdoMet via π‐stacking interactions between its indole group and the adenine group of AdoMet, as has been suggested to occur for Efm6 23. These relative positions of the tryptophans are also observable in the structures of METTL21A‐D and CaM‐KMT (Fig. S3). Overall, this strongly suggests an important role for these tryptophans in the activity of Efm2 and in Family 16 methyltransferases.

**Figure 2:**
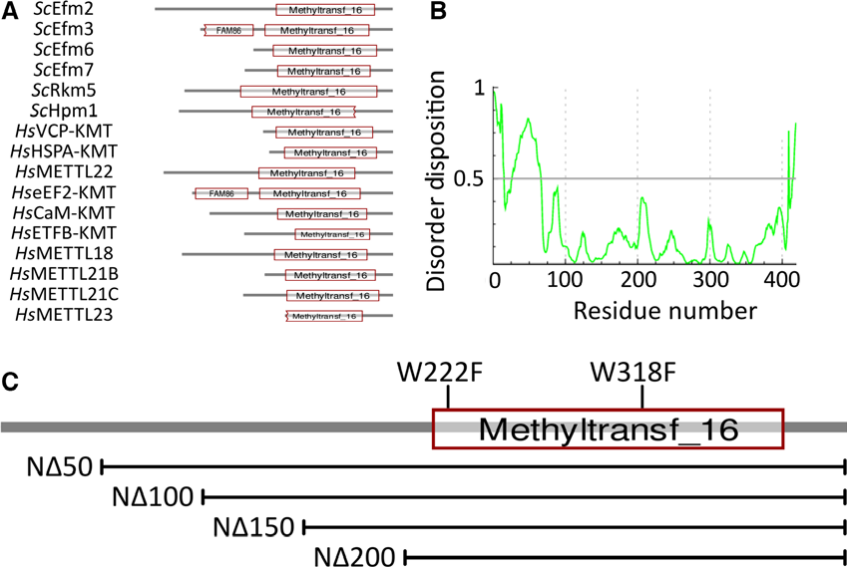
Efm2 has a long, partially disordered N‐terminal region separate from the core methyltransferase domain. (A) The domain architectures of all known Family 16 methyltransferases from yeast and human. A number of them have extended N‐terminal regions; Efm2 has the longest of all. Methyltransferase lengths are to scale; Efm2 is 419 residues long. Jagged edges of domains represent partial truncations. (B) The predicted disorder of Efm2 as determined by pondr‐fit. Disorder disposition > 0.5 represents disorder. (C) Mutagenesis of Efm2. Four N‐terminal truncation mutants and two point mutants were generated in order to characterise the N‐terminal region and the conserved tryptophans in Efm2. Note that all four N‐terminal truncations retain the core Family 16 methyltransferase domain.

To investigate the prevalence of N‐terminal extensions beyond the core Family 16 methyltransferase domain, we visualised the domain architecture of all yeast and human Family 16 methyltransferases using CDvist (Fig. 2A). This showed that Efm2 has the longest N‐terminal extension. Interestingly, some of these methyltransferases, notably Efm6, Efm7, VCP‐KMT and HSPA‐KMT, have very short N‐terminal extensions, in sharp contrast with Efm2. The N‐terminal ~ 50 residues of Efm2 are predicted to be disordered, with the rest of the protein being predominantly ordered, as typified by the prediction made by Pondr‐fit (Fig. 2B).

We therefore sought to investigate the importance of the two conserved tryptophans and N‐terminal region in Efm2. We generated conservative tryptophan‐to‐phenylalanine point mutations for W222 and W318 and four N‐terminal truncations, as depicted in Fig. 2C, and investigated their methyltransferase activities compared to the wild‐type enzyme.

### Conservative point mutations indicate the importance of two key tryptophan residues

**Figure 3:**
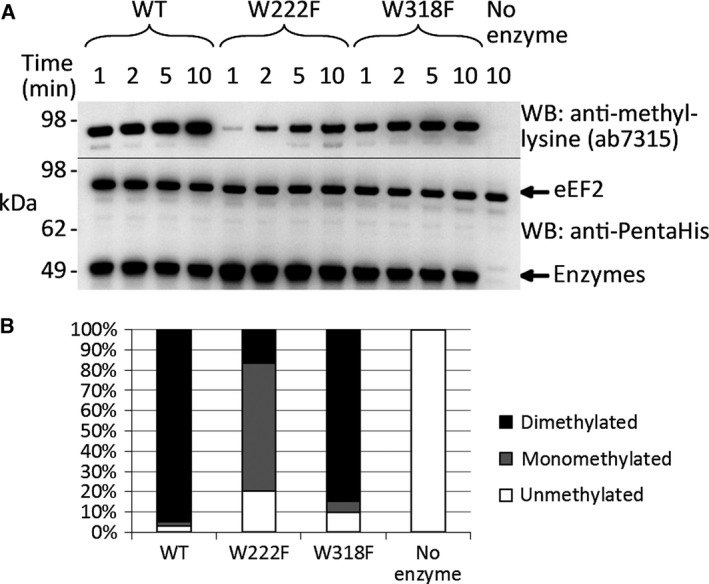
Tryptophan point mutants of Efm2 show reduced methyltransferase activity. eEF2 was incubated with wild‐type (WT), W222F or W318F Efm2 in the presence of AdoMet for 1, 2, 5 or 10 min, before reactions were resolved by SDS/PAGE and analysed by immunoblotting (A) or LC‐MS/MS (B). No enzyme was added for a negative control. (A) Immunoblotting with the anti‐methyl‐lysine antibody ab7315 (top) revealed markedly reduced methyltransferase activity of the W222F mutant, while the W318F mutant showed slightly reduced activity. An immunoblot with the anti‐PentaHis antibody was used as a loading control (bottom). (B) LC‐MS/MS of eEF2 from the 10‐min assay revealed that the wild‐type enzyme was able to produce almost complete dimethylation of K613 in the peptide DDFKAR, while the W222F mutant produced predominantly monomethylation and the W318F mutant produced nearly the same level of dimethylation as the wild‐type enzyme. The no enzyme control did not show any gain of methylation. The relative amount of each methylation state was determined by the area under the curve for each peak in the extracted ion chromatograms (XICs), which are shown in Fig. S4. W222 therefore appears to be important for methyltransferase activity, while W318 is not as important.

Given that the tryptophans are likely to be critical for methyltransferase activity, we mutated them to the similarly hydrophobic and aromatic residue, phenylalanine. Immunoblotting of a time‐series assay of methyltransferase activity on eEF2, compared to wild‐type Efm2, showed a severe reduction in methyltransferase activity with the W222F mutant, while the W318F showed only a slight reduction in activity (Fig. 3A). LC‐MS/MS analysis of eEF2 from the 10‐min time‐point showed that wild‐type Efm2 produced predominantly dimethylation of K613 in the peptide DDFKAR (Fig. 3B). The W318F mutant produced slightly less dimethylation than the wild‐type, and had slightly more unmethylated eEF2 remaining (Fig. 3B). The W222F mutant, however, produced predominantly monomethylation (Fig. 3B). This could reflect a decreased rate of activity or a change in the degree of methylation the enzyme is capable of catalysing. The pronounced effect of the W222F mutation may be due to the proximity of W222 to the active site of Efm2, as mentioned above. The fact that the W318F mutant had an activity comparable to that of the wild‐type enzyme indicates either that W318 is not critical for binding AdoMet, or that the phenylalanine is sufficiently similar to the structure of tryptophan to bind AdoMet effectively.

### Efm2 N‐terminal truncations reveal the importance of the N terminus for methyltransferase activity

**Figure 4:**
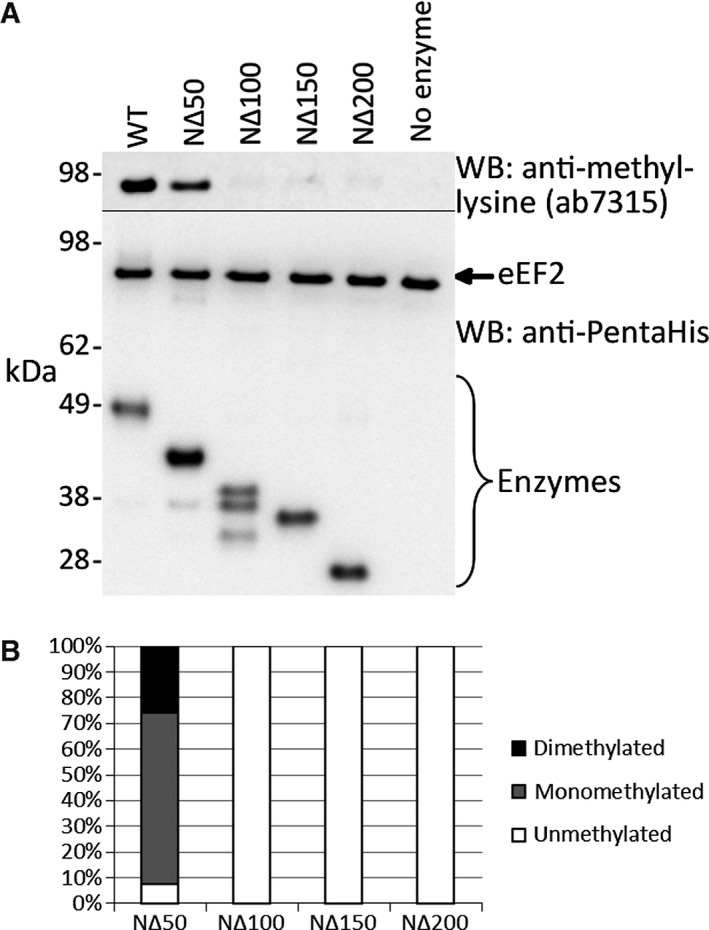
N‐terminal truncations of Efm2 show severely reduced methyltransferase activity. eEF2 was incubated with wild‐type (WT) or N‐terminally truncated Efm2 (NΔ50, NΔ100, NΔ150 and NΔ200) in the presence of AdoMet for 1 h, before reactions were resolved by SDS/PAGE and analysed by immunoblotting (A) or LC‐MS/MS (B). No enzyme was added for a negative control. (A) Immunoblotting with the anti‐methyl‐lysine antibody ab7315 (top) revealed markedly reduced methyltransferase activity of the NΔ50 mutant, while the NΔ100, NΔ150 and NΔ200 mutants showed no activity. An immunoblot with the anti‐PentaHis antibody was used as a loading control (bottom). (B) LC‐MS/MS of eEF2 revealed that the NΔ50 mutant produced predominantly monomethylation of K613 in the peptide DDFKAR, while the NΔ100, NΔ150 and NΔ200 mutants produced no methylation, in agreement with the immunoblot. The relative amount of each methylation state was determined by the area under the curve for each peak in the extracted ion chromatograms (XICs), which are shown in Fig. S5. The N terminus of Efm2, from residue 50 onwards, is therefore critical for its methyltransferase activity.

We investigated the unique extended N‐terminal region of Efm2 by generating four incremental 50 amino acid N‐terminal truncations (Fig. 2C) and testing their methyltransferase activity on eEF2 over 1 h. Surprisingly, immunoblotting suggested that the NΔ100, NΔ150 and NΔ200 truncations all showed no methyltransferase activity, while the NΔ50 truncation showed reduced activity compared to wild‐type Efm2 (Fig. 4A). LC‐MS/MS of eEF2 (Fig. 4B) confirmed the lack of activity observed for NΔ100, NΔ150 and NΔ200 and showed that NΔ50 produced predominantly monomethylation at K613 in the peptide DDFKAR, and markedly less dimethylation than wild‐type Efm2 produces in 10 min (see above). Together, these results indicate that the N‐terminal region of Efm2, particularly residues 50–200, is important for its methyltransferase activity.

**Figure 5:**
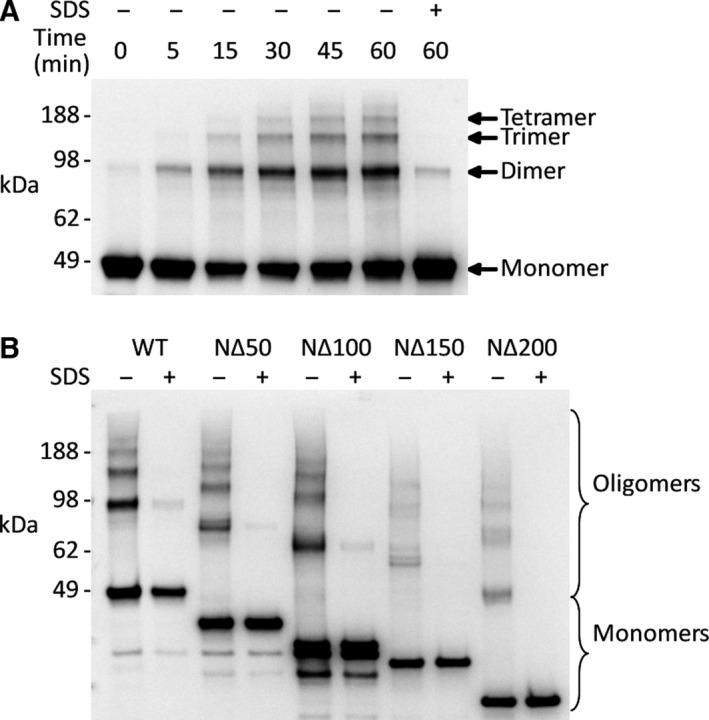
Efm2 and its N‐terminal truncation mutants form oligomeric states. Wild‐type (WT) Efm2 and its N‐terminal truncation mutants were incubated with or without SDS in the presence of the crosslinker dimethyl pimelimidate. An anti‐PentaHis immunoblot was used to detect all relevant species. (A) Time‐series crosslinking analysis of wild‐type Efm2 indicates that it forms oligomeric states. (B) Crosslinking of Efm2 N‐terminal truncation mutants for 2 h. SDS (1%) added prior to crosslinking was used as a negative control for all N‐terminal truncations.

One possible explanation for this loss of methyltransferase activity is that the N‐terminal region is required for Efm2 to self‐interact and form oligomers necessary for its activity, as is the case for some other protein methyltransferases 46, 47. In order to investigate this possibility, we first tested whether wild‐type Efm2 is capable of forming oligomers. *In vitro* chemical crosslinking followed by SDS/PAGE revealed that wild‐type Efm2 forms a series of oligomeric states, as bands corresponding to the dimer (~ 96 kDa), trimer (~ 144 kDa) and tetramer (~ 192 kDa) were seen to gradually form over an hour of crosslinking (Fig. 5A). A negative control with 1% SDS added prior to crosslinking showed minimal formation of oligomeric bands, indicating that the crosslinking is due to the self‐interaction of Efm2, which is disturbed upon denaturation by SDS. We then tested the ability of the four N‐terminal truncations to form oligomers after 2 h of crosslinking, compared to wild‐type Efm2 and SDS controls. This showed that all four N‐terminal truncations were able to form the oligomeric states observed for wild‐type Efm2 (Fig. 5B). This indicates that the N‐terminal 200 residues of Efm2 are not critical for oligomerisation, and suggests that the effect on methyltransferase activity observed upon N‐terminal truncation may be due to a loss of interaction with its substrate eEF2.

## Discussion

Elongation factor methyltransferase 2 is one of the Family 16 group of methyltransferases, a group of newly discovered eukaryotic enzymes that all methylate nonhistone proteins. Here we have investigated Efm2 by site‐directed mutagenesis and truncation in order to better understand Family 16 methyltransferases. We have shown that one completely conserved tryptophan (W222 in Efm2) is important for methyltransferase activity, while the other, which is present in all but one Family 16 methyltransferase, is of lesser consequence. This disparity may be explained by the fact that W222 is positioned at the active site, while W318 is not. The shift to monomethylation of K613 in eEF2, seen with the W222F mutation of Efm2, could indicate that W222 determines the degree of methylation by affecting the size of the active site and thus how many methyl groups can be added to a substrate. The SET domain methyltransferases use a similar mechanism to control the degree of substrate methylation 48. It could alternatively be that the W222F mutation reduced the rate of activity of Efm2, and thus the monomethylation was simply an intermediate in the formation of dimethylation. This would point to a distributive mechanism of action, as Efm2 would dissociate from eEF2 after the formation of monomethylation.

**Figure 6:**
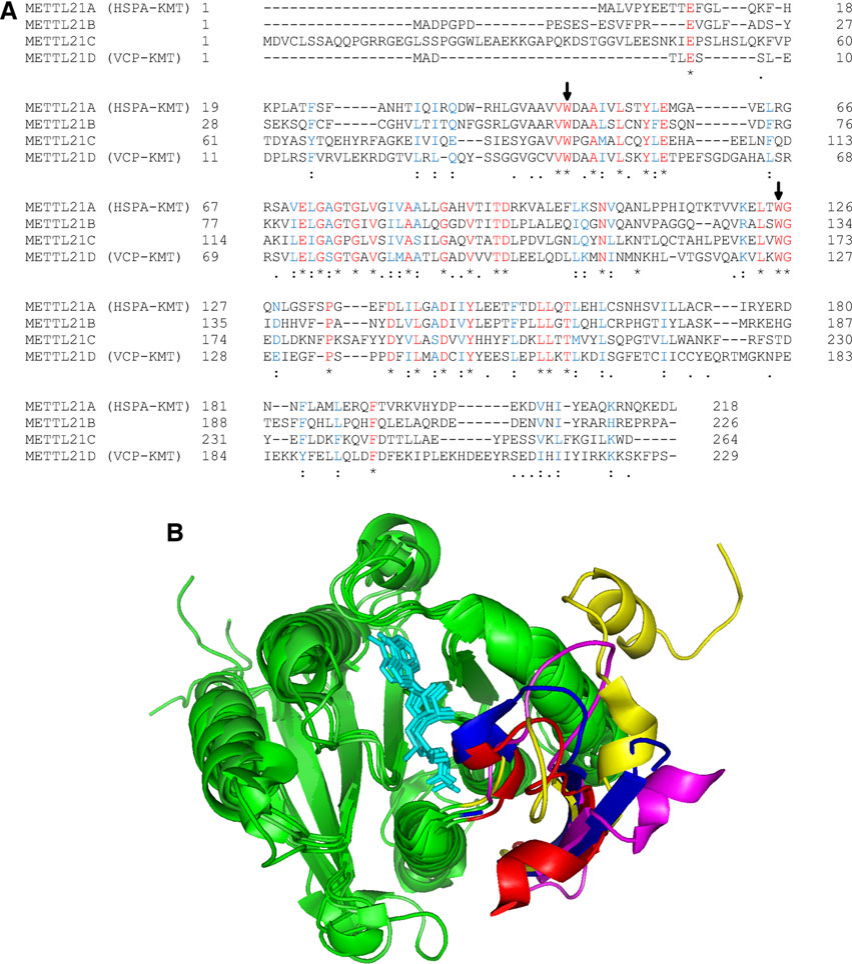
The human METTL21 proteins exemplify the variability of N‐terminal regions of Family 16 methyltransferases. (A) Alignment of human METTL21A (HSPA‐KMT), METTL21B, METTL21C and METTL21D (VCP‐KMT) using Clustal Omega with default settings 36. Asterisks (*) and red colour indicate identical residues; double dots (:) and blue colour indicate chemically similar residues; single dots (.) indicate dissimilar residues; dashes (–) indicate missing residues. The two conserved tryptophans are indicated with arrows. (B) Alignment of the structures of the METTL21s (PDB IDs: 4LEC, 4QPN, 4MTL, 4LG1), showing their highly similar core folds (green, AdoMet in cyan) and their highly dissimilar N‐terminal regions (residues 15–46 of METTL21A: red; residues 24–56 of METTL21B: blue; residues 45–91 of METTL21C: yellow; residues 11–42 of METTL21D: magenta). Visualised in pymol (The PyMOL Molecular Graphics System, Version 1.3 Schrödinger, LLC).

We have also shown that the N‐terminal 50–200 residues of Efm2 are essential for its methyltransferase activity. Interestingly, this correlates with the predicted structural order of this region of Efm2 from residue ~ 80 onwards. This suggests that the N‐terminal region of Efm2 may form tertiary structures that are critical for its specific recognition of eEF2; it also raises a broader possibility that the N‐terminal regions of Family 16 methyltransferases are critical for their specificity. This is best exemplified by the human METTL21 proteins (METTL21A‐D), which form a subgroup within Family 16 methyltransferases due to their similarity (Fig. 6A). METTL21A (HSPA‐KMT) and METTL21D (VCP‐KMT) methylate different proteins, while METTL21B and METTL21C have no known substrates. Alignment of the crystal structures of all four of these proteins demonstrates the remarkable similarity of their core methyltransferase domains, while revealing highly variable N‐terminal regions (Fig. 6B). It is therefore likely that these N‐terminal regions of the METTL21 proteins, and Family 16 methyltransferases in general, are important for determining their substrate protein specificity.

Efm2 was shown to form a number of oligomeric states. Several protein methyltransferases have been previously shown to form oligomers, including the arginine methyltransferases Hmt1 46, CARM1 49, PRMT1 47, PRMT3 50, 51, PRMT5 52, PRMT8 53 and some lysine methyltransferases such as G9a, GLP 54 and SU(VAR)3‐9 55. The oligomerisation of Efm2 may point to a processive mechanism of action, as a dimer of Efm2 could achieve dimethylation without detachment from eEF2, as is the case for many arginine methyltransferases 56. This is the first demonstration that a Family 16 methyltransferase forms oligomers, and it would be interesting to investigate whether other Family 16 methyltransferases also oligomerise.

While Family 16 methyltransferases have only been discovered recently, their biological and medical significance is already becoming apparent. Efm2 and Efm3 both act on eEF2 in yeast, and deletion of either methyltransferase has been shown to increase sensitivity to translational inhibitors 21, 22. Both were also shown to have potential effects on translational fidelity 21, 22. In human, VCP methylation by VCP‐KMT appears to affect its ATPase activity and the methyltransferase was shown to be important for cell migration and invasion, suggesting a role in cancer metastasis 28, 29. METTL22‐mediated methylation of Kin17 appears to regulate its association with chromatin 31, while CaM‐KMT appears to be important for normal body growth and somatosensory development 57. HSPA‐KMT‐mediated methylation of HSPA8 was shown to reduce its affinity for α‐Syn, a protein whose aggregation is associated with Parkinson's disease 30. HSPA1 methylation, also catalysed by HSPA‐KMT, was recently found to correlate with cancer outcome 58. Even some Family 16 methyltransferases without known substrates have been associated with disease. METTL21B has been linked to multiple sclerosis 59, 60, 61, mutation of METTL23 has been linked to intellectual disability 62, 63 and METTL21C has been found to be important in muscle cell differentiation and bone cell viability, and thereby is associated with osteoporosis and sarcopenia 64. It will therefore be of great interest to discover the targets of these methyltransferases, and to further characterise these and all other Family 16 methyltransferases, in order to better understand their biological functions and roles in disease.

## Author contributions

JJH and MRW conceived of the study. JJH, MAE and GHS performed experiments. JJH and MRW drafted the manuscript, with input from all other authors.

## Supporting information


**Fig. S1.** Example spectra showing mono‐ and dimethylation of K613 in peptide DDFKAR from eEF2.

**Fig. S2.** Full sequence logo of human and yeast Family 16 methyltransferases.

**Fig. S3.** Structures of Family 16 methyltransferases show identical placement of conserved tryptophans.

**Fig. S4.** Extracted ion chromatograms for Fig. 3B.

**Fig. S5.** Extracted ion chromatograms for Fig. 4B.
