# Supplementary figures and images for: The activity of a yeast Family 16 methyltransferase, Efm2, is affected by a conserved tryptophan and its N‐terminal region
Source: FEBS Open Bio. 2016 Nov 16;6(12):1320–30. doi: 10.1002/2211-5463.12153 (PMC5324768; doi:10.1002/2211-5463.12153)

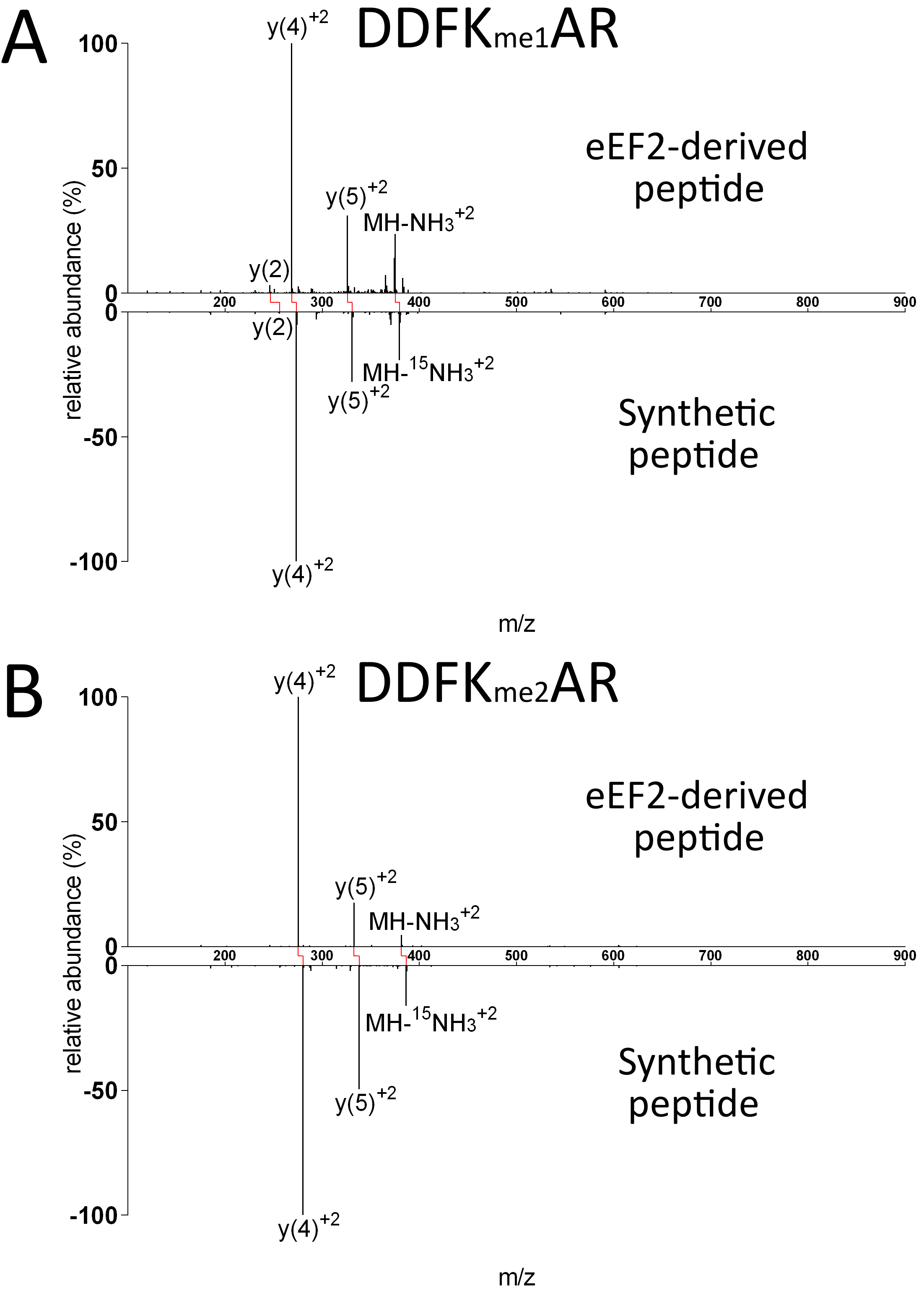

Supplement: Supplementary file 1 — Fig. S1. Example spectra showing mono‐ and dimethylation of K613 in peptide DDFKAR from eEF2. [file FEB4-6-1320-s001.tiff]

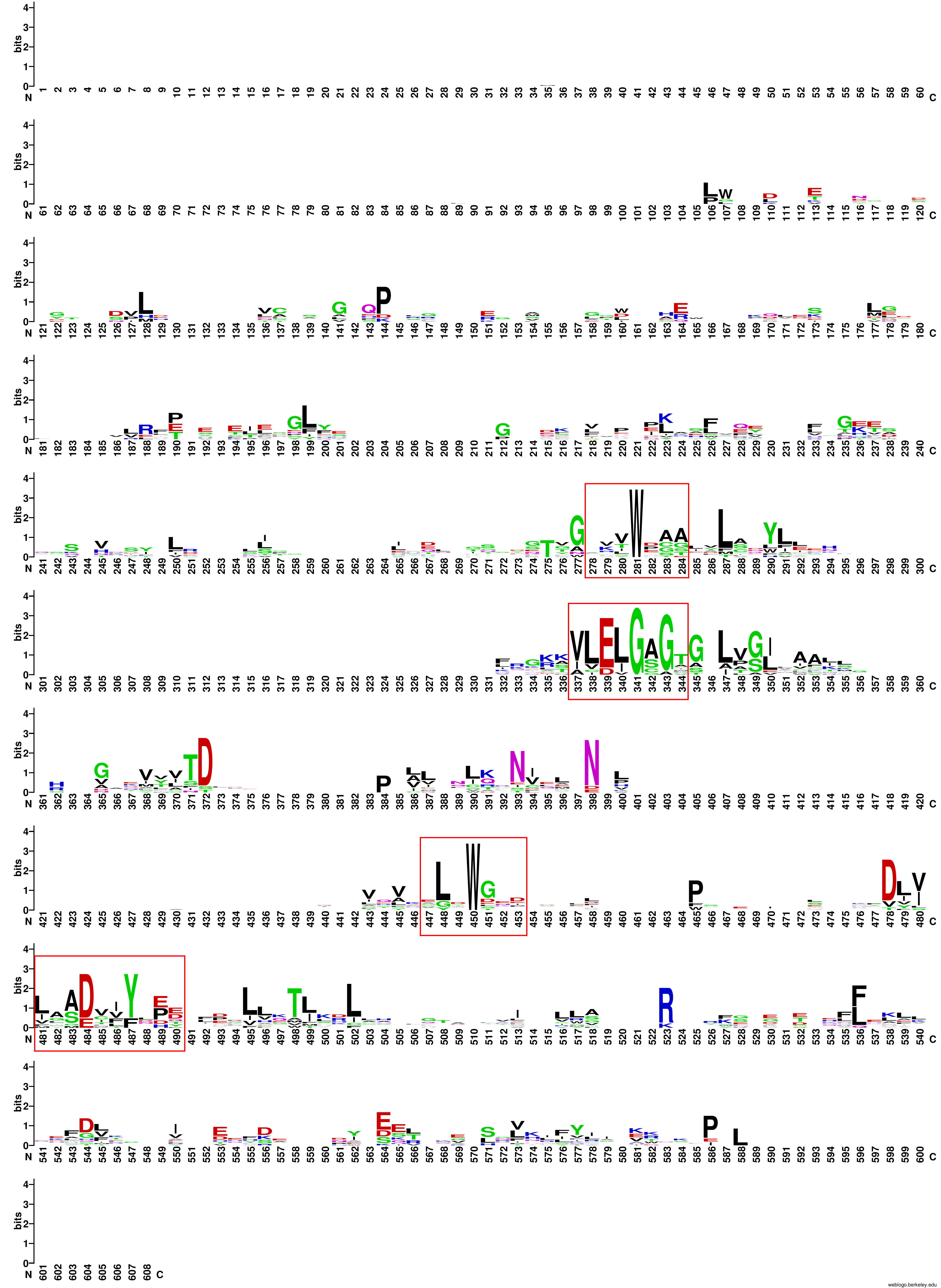

Supplement: Supplementary file 2 — Fig. S2. Full sequence logo of human and yeast Family 16 methyltransferases. [file FEB4-6-1320-s002.tif]

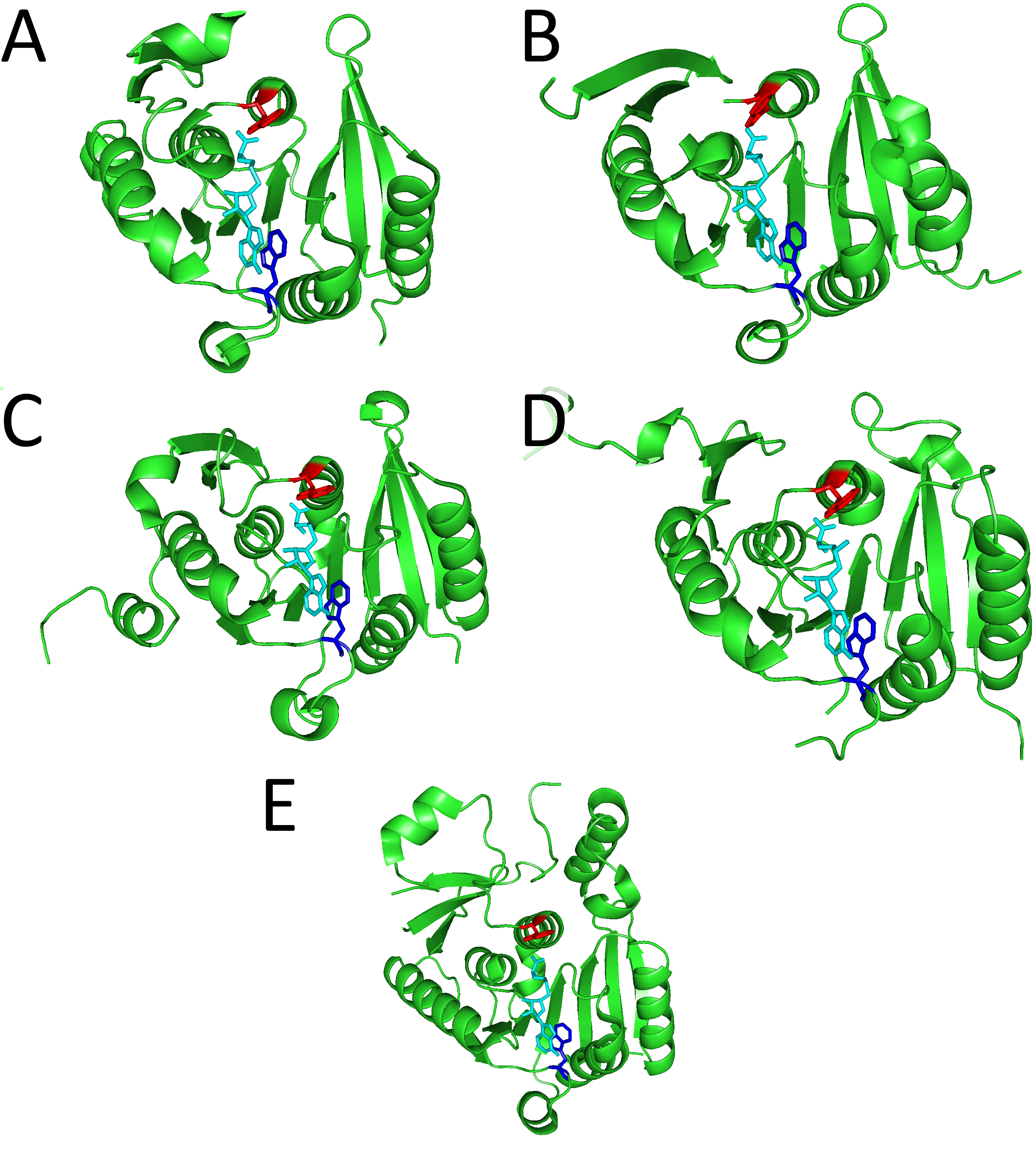

Supplement: Supplementary file 3 — Fig. S3. Structures of Family 16 methyltransferases show identical placement of conserved tryptophans. [file FEB4-6-1320-s003.tif]

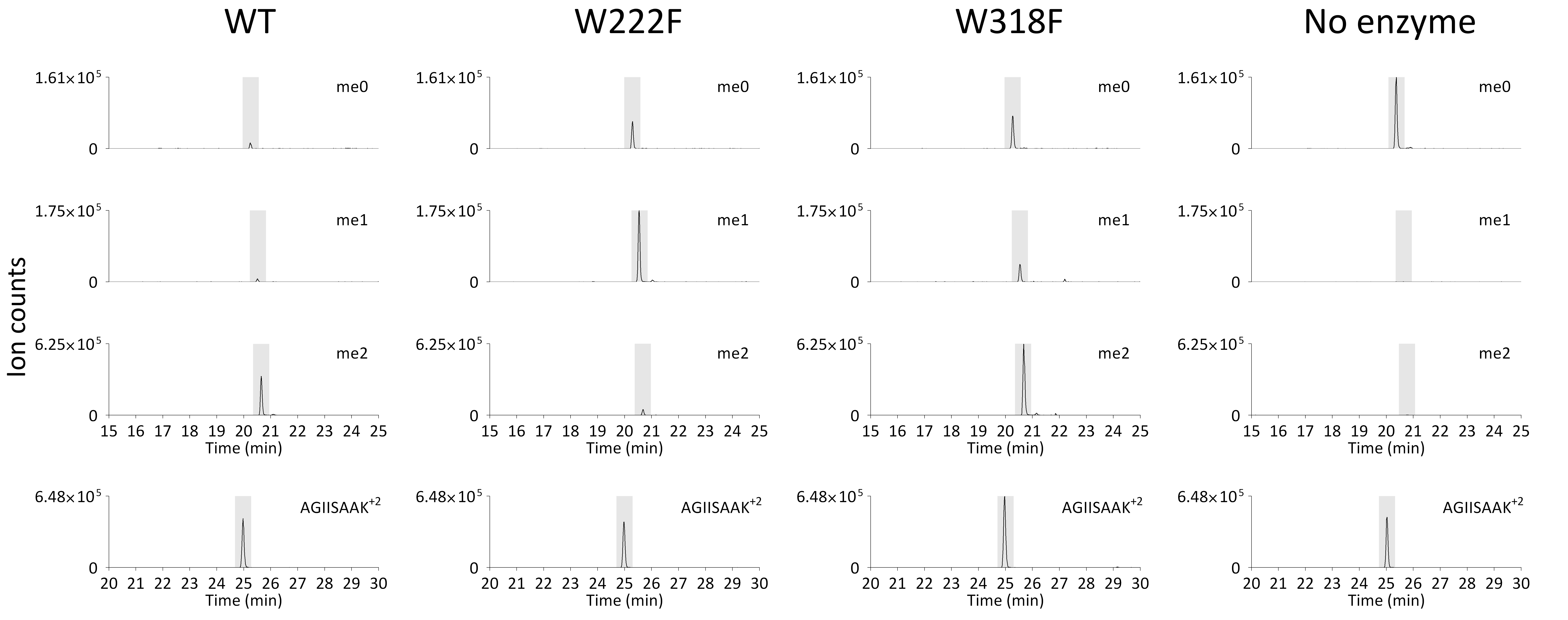

Supplement: Supplementary file 4 — Fig. S4. Extracted ion chromatograms for Fig. 3B. [file FEB4-6-1320-s004.tif]

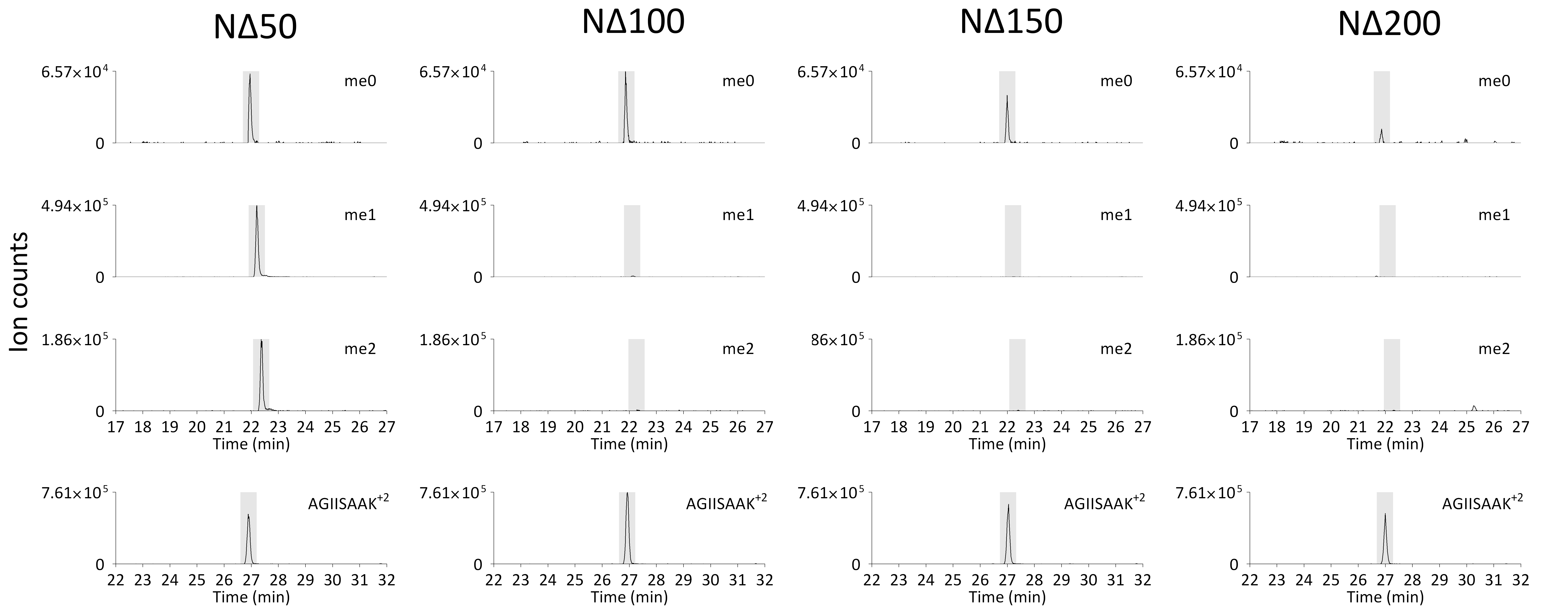

Supplement: Supplementary file 5 — Fig. S5. Extracted ion chromatograms for Fig. 4B. [file FEB4-6-1320-s005.tif]
